# Surgical debridement of infected pubic symphysitis supports optimal outcome

**DOI:** 10.1007/s00402-020-03563-8

**Published:** 2020-08-14

**Authors:** Benjamin Devlieger, Daniel Wagner, Johannes Hopf, Pol Maria Rommens

**Affiliations:** Department of Orthopaedics and Traumatology, University Medical Center Mainz, Mainz, Germany

**Keywords:** Pubic symphysis, Symphysitis, Diagnosis, Debridement, Infection

## Abstract

**Introduction:**

Infected pubic symphysitis (IPS) is a rare bacterial infection of the pubic symphysis that causes subpubic pain, disability and ultimately permanent immobility. Due to difficult diagnosis, patients present with long-standing complaints and consult several doctors. To date, no validated treatment protocol exists and most patients are treated conservatively with antibiotics. This study was aimed to assess the results after careful surgical debridement and pathogen-specific antibiotic treatment in IPS.

**Materials and methods:**

A chart review of eight patients with proven IPS was performed. Five of eight patients filled in a specific own-developed questionnaire and could be examined clinically and radiologically at a mean of 13 months (range: 6–30 months) postoperatively.

**Results:**

There were six males and two females with an average age of 69 years (range: 55–80 years). The mean duration of symptoms before surgical treatment was 10.5 months (range: 1–30 months). There were no complications due to the surgical debridement. There was no recurrence of infection at the pubic symphysis during the follow-up period. The most common pathogen was *Pseudomonas aeruginosa* in three patients. Mean preoperative pain, measured on the visual analogue scale (VAS, range: 0–10) for the four analysed categories in the five follow-up patients was 7.2, 30 days postoperatively 2.7 and 13 months postoperatively 0.4. There was a steady increase in the quality of life (QoL) 30 days postoperatively and at the 13 months follow-up when compared to preoperative values.

**Conclusions:**

Surgical debridement is the keystone for treatment of IPS and should be combined with local and systemic antibiotic therapy.

## Introduction

Infected pubic symphysitis (IPS), a bacterial infection of the symphysis pubis, is a rare condition that ultimately causes disability, hospitalization and may require emergent therapy. To date, only about 200 cases have been described [1, 2]. IPS presents with long-standing anterior pelvic pain or pubalgia. Symphysis-related pubalgia can however also be caused by pubic osteitis, its more common aseptic counterpart [3, 4]. In both entities, patients present with pain on ambulation, severe pubic and groin discomfort and gait instability; however, only IPS can also present with fever [5]. IPS differs from aseptic osteitis pubis primarily through its infectious origin, making bacteriology the only true differentiator. This causes a confusion of data, where beginning IPS can be misdiagnosed as osteitis pubis when no bacteriology is taken. The causes of IPS are multifarious, representing 2% of haematogenous osteomyelitis cases [6] on one hand and resulting after traumatic events or surgery on organs of the small pelvis on the other [5, 7, 8]. In contrast, aseptic osteitis pubis is most likely caused by periosteal trauma [9] and is often seen in athletes as a progression of sports hernia, although a variety of aetiologies exist [10]. Other differential diagnoses of pubic pain include urologic, gynaecologic and various musculoskeletal diseases [11–13]. Differentiating between IPS and osteitis pubis can thus be challenging and often requires a detailed patient history, clinical examination and complementing imaging and ultimately collecting bacteriology specimens. Optimal treatment is still open to discussion, with expert opinion unclear on when to operate, prolonging patient discomfort and disability. To our knowledge, no study exists describing the technique and results of surgical debridement in combination with local and systemic antibiotic therapy in proven IPS. In this manuscript, the authors present the technique of debridement and the results of surgical and antibiotic therapy in eight patients.

## Patients and methods

### Diagnostic workup

Patients were admitted for further diagnostic examination when presenting with clinical signs of infection, such as local swelling, increased heat, local tenderness on pressure and immobilizing pain when attempting to walk. Routine blood results such as C-reactive protein and leucocyte count were obtained and compared to previous results to determine the trend of infectious markers. Pelvic radiographs were taken to exclude other musculoskeletal disorders. If no recent diagnostic imaging was performed, a pelvic CT and MRI without contrast were both performed to identify the extent of bony and soft tissue pathology around the pubic symphysis. On pelvic CT, especially destructive erosions of the bony margins were searched for, and on pelvic MRI the presence of a fluid collection inside and around the pubic symphysis was looked at. Both CT and MRI are needed to support or discourage operative therapy. The decision to perform surgical debridement was made by a multidisciplinary board of surgeons and radiologists, considering the severity of the symptoms, the laboratory examinations and correlation to the CT and MRI images. The diagnostic modalities of IPS are described in more detail in the Appendix.

### Operative technique

The patient was positioned supine on a radiolucent operation table. Preoperative bladder catheterization is critical to avoid iatrogenic penetration of a protruding bladder obstructing the surgical approach. Surgical debridement was performed through a Pfannenstiel incision or through the incision of previous surgeries. Often, the local anatomic landmarks become unclear through adhesions. In these cases, the pubic symphysis was located with a small-bore needle using a fluoroscopic image in the AP direction. Special attention was paid to the preservation of the integrity of the pubic ligaments. The symphysis was opened through an incision of the superior ligaments only. When the joint was already open through the infection, no further incision of the ligaments was performed. With curettes of increasing diameter, the whole depth of the pubic symphysis was debrided and cleaned carefully avoiding any penetration. Several fluid and tissue samples were taken for microbiologic cultures. The joint was extensively irrigated. Local absorbable antibiotic carriers (50 mg gentamicin sulphate, Sulmycin Implant E, EUSA Pharma Europe) were placed in the joint and the different tissue layers separately closed.

### Study design

We retrospectively searched the medical charts of all patients in a level I trauma centre for microbiologically proven IPS, who were treated with surgical debridement in combination with local and systemic antibiotic therapy in our department. After the study was approved by the local ethical committee,^1^ we explored our database using multiple case-attributed diagnostic and operative codes.^2^ No date-limit was set for the period of inclusion. The found files (*n* = 166) were individually scanned to exclude patients without pubic pathology and 13 patients with symphysis-related pubalgia were identified. Three patients with less severe symptoms were treated conservatively with analgesia and physiotherapy as osteitis pubis (one outpatient, two inpatients). Two were excluded from analysis due to the presence of a chronic fistula, one of which necessitated surgical debridement and the other treated with oral antibiotics. The decision to exclude certain patients from analysis was made a priori, accepting a reduction in study power to be able to describe a homogeneous patient population. The eight remaining patients with proven IPS were included in the analysis. Included patients were contacted by telephone and invited for a clinical and radiological follow-up examination. The patients were asked to fill in an own-developed questionnaire, which consisted of scale-response questions covering three topics: pain, mobility and quality of life. Each topic was measured on three different times: preoperative, 30 days postoperatively and at the time of follow-up examination (Table 1). Pain was documented on the visual analogue scale (VAS, range 0–10) in different situations (supine, seated, standing, and walking). The own-developed quality of life (QoL) questionnaire measured quality of sleep, dependence on others and nursing staff, psychological disturbances and ability to perform daily tasks with a minimum value of 1 (worse) and a maximum of 5 (best). All values were summated and averaged for the three questioned times. Additionally, patients were asked if they had a recurrence of infection after treatment. Satisfaction was noted on the same scale used in the QoL questionnaire with a minimum value of 1 (worse) and a maximum of 5 (best). Clinical examination consisted of inspection, palpation and mobility testing. The mobility tests were an examination of gait, hip mobility and the registration of a limp hump and/or Trendelenburg sign. The results of the clinical examinations were recorded on a checklist. Comorbidities and additional pathology were noted separately. In the absence of conclusive diagnostic tools, we developed a diagnostic checklist that lists the important clinical findings for diagnosis of IPS (Table 2).

**Table 1:** Contents of the questionnaire of every patient (*n* = 5) filled in at the follow-up

Questionnaire	
Date of first symptoms Pain	VAS in different situations (0–10) Supine Seated Standing Walking
Quality of life	Bad till optimal (1–5) Quality of sleep Dependence on others and nursing staff Psychological disturbances Ability to perform daily tasks Subjective quality of life
Mobility	Walking aids needed (Y/N and which needed) Maximum walking distance with and without walking aids Mobility in own home possible (Y/N)
Recurrence (Y/N)	Recurrence of infection after treatment
Satisfaction (0–5)	Overall satisfaction regarding operative procedure and result
Other remarks	Open response

**Table 2:** Summary of important diagnostic information on IPS, including what to ask, what to look for and which examinations are necessary

Key preoperative diagnostic information of IPS
History Recent pelvic trauma Previous pelvic surgery Recurrence of symptoms after interruption of antibiotic therapy Impact of pelvic pain on QoL
Clinical Tenderness of the pubic symphysis Pain during walking leading to loss of mobility
Inflammation markers CRP or Lc increase
Imaging Osteolysis of the symphysis on conventional X-ray or CT imaging High signal intensity in the pubic joint space on T2-weighted and low signal intensity on T1-weighted images on MRI images

## Results

Eight patients were included with microbiologically confirmed infection and received surgical debridement. One patient died due to liver failure during the follow-up period. One patient was not available because of ongoing chemotherapy, and another patient was unavailable due to an unrelated geriatric hospital admission. Five of eight patients were available to answer the questionnaire and undergo a clinical and radiological follow-up examination.

### Case presentation (Fig. 1a–d)

**Fig. 1:**
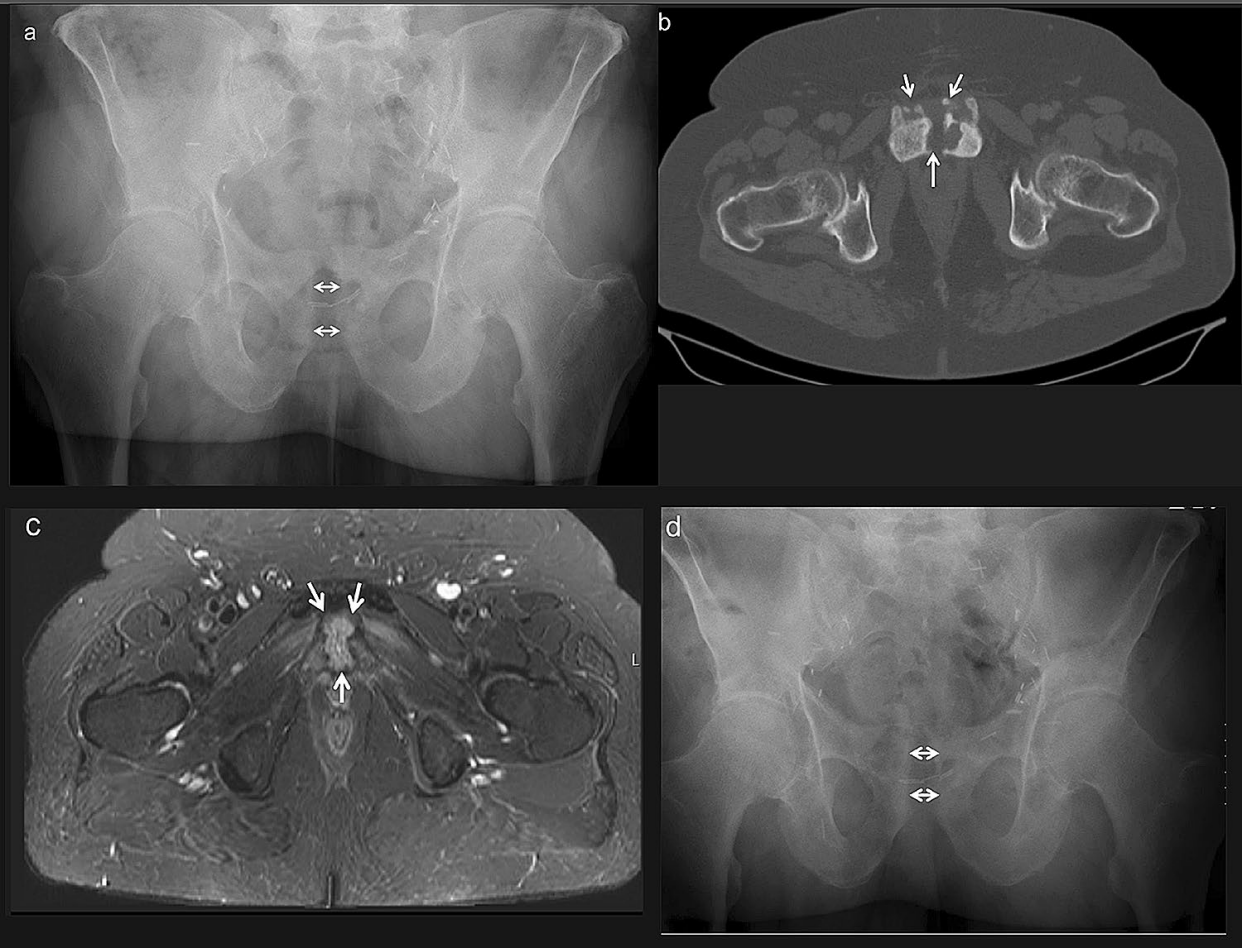
**a**–**d** A 69-year-old male with infected pubic symphysitis. Preoperative anteroposterior radiograph of the pelvis showing a slight widening of the pubic symphysis (arrows). Surgical clips of the previous prostatectomy are visible on both sides (**a**). CT slice of the anterior pelvis shows irregularities of the bony surfaces and necrotic bone fragments in the pubic symphysis joint space (arrows) (**b**). T2-weighted MRI transection of the pubic symphysis shows a fluid collection in and behind the joint space (arrows) (**c**). Postoperative anteroposterior radiograph of the pelvis with similar widening of the pubic symphysis as preoperative (arrows) (**d**)

A 69-year-old, retired male nurse presented with pubic pain, increased during walking. After a cystoprostatectomy, he had constant pubic pain during 3 consecutive years. His body mass index was 39. The cystoprostatectomy had been complicated by a surgical site infection with *Pseudomonas aeruginosa* that was treated with vacuum-assisted closure (VAC) therapy and systemic antibiotics for 4 weeks. Three weeks before the presentation in our department, pain over the old operation scar had become more intensive. His walking distance was limited to 200 m. Previous treatment with ibuprofen, paracetamol and metamizole had provided no benefit. A widened scar over the symphysis was observed with pubic tenderness. There was no inguinal tenderness with a free range of motion of the hips. A previously performed Tc99 white blood cell scintigraphy showed no increased signalization at the symphysis. Plain radiographs of the pelvis, which were taken at the time of his first presentation in our department, showed a widening of the pubic symphysis (Fig. 1a). A CT scan showed bone irregularities and necrotic bone fragments in the pubic symphysis joint space (Fig. 1b). T2-weighted MRI transections revealed a fluid collection within and behind the pubic symphysis joint space (Fig. 1c). Laboratory tests showed a C-reactive protein (CRP) level of 9.7 mg/L and a leucocyte count (Lc) of 9.01/µL. The patient was admitted, and surgical debridement was performed as described above. *Pseudomonas aeruginosa* was cultured from the surgical specimens. The patient received intravenous antibiotics for 10 days with clindamycin and was released with a 6-week regimen of oral clindamycin. Follow-up at 6 weeks revealed pain-free walking and no tenderness of the symphysis or the pelvic ring. Between week 6 and 12, the patient was re-hospitalized in our urology department for 3 weeks due to a urinary tract infection with *Escherichia coli*. The 3 months follow-up showed no remaining symptoms of the pubic symphysis. The clinical followup described above was performed 10 months postoperatively. He had no pain for the four activities and no mobility restrictions at a reported walking distance of 2 km. His mean QoL for the questioned areas was 4.6/5. The plain radiograph of the pelvis after 10 months showed no further widening of the pubic symphysis (Fig. 1d).

### Demographics/chart review (*n* = 8)

There were six male and two female patients with a mean age of 69 years (range: 55–80 years). All had spontaneous pubic pain with aggravation during walking. The presentation in our department was after a mean of 10.5 months after the onset of pubic pain (range: 1–30 months, median 3.5 months). No patient had fever lasting more than 6 h. All patients had consulted several doctors for the same complaints before presentation in our department. Seven patients had a history of surgery in proximity of the symphysis: four underwent a prostatectomy, one hernia repair, one hysterectomy, and one ileal conduit bladder diversion. All patients had an American Society of Anaesthesiologists (ASA) score of either 2 or 3. Inflammatory markers showed a wide range with a mean CRP of 60 mg/dL (range: 1-227 mg/dL) and a mean Lc of 9.27/µL (range: 9.2–15.2/µL). CRP values were elevated in patients presenting early after onset of symptoms, whereas low to normal CRP levels were seen in patients with delayed presentation. Due to delayed presentation, patients presented with a variety of medical imaging workup, often consisting of a triad of pelvic X-rays, pelvic CT Imaging and pelvic MRI scans. On conventional X-rays, osteolysis of the pubic bones adjacent to the symphysis was seen in all but one patient. Further information on diagnostic measures and patient characteristics are depicted in Table 3. During surgical exploration, abscess formation was not found. In contrast, extensive scar tissue was present in all patients. A clear or slightly cloudy fluid was found in the joint space. Infected tissue was debrided, specimens were sent to microbiology and a local antibiotic sponge was inserted in the joint. Postoperatively, all patients were treated with empiric intravenous antibiotics. The antibiotics were adjusted to the sensitivity of the germs during the hospital stay and continued orally after discharge. A single pathogen could be isolated in all patients. *Pseudomonas aeruginosa* was the most common germ, affecting three patients (Table 4). The mean length of hospital stay was 21 days (range: 6–86 days). No operative revision of the pubic symphysis was necessary during the hospital stay. During the follow-up period, one patient died due to liver failure. Table 3 shows further details on the postoperative course.

**Table 3:** Patient characteristics of the patients included for analysis (*n* = 8)

Patient characteristics (*n* = 8)			
	Number	Mean	Median
Patient characteristics			
Age		69	71
M/F	6/2		
BMI		30.8	30.3
Weight (kg)		92	85
Height (cm)		172	173
Treatment			
Intravenous antibiotics (weeks)	1–2		
Oral antibiotics (weeks)	2–8		
Postoperative complications	0		
Length of hospital stay (days)	6–86	21.7	14.0
Mortality	1

**Table 4:**
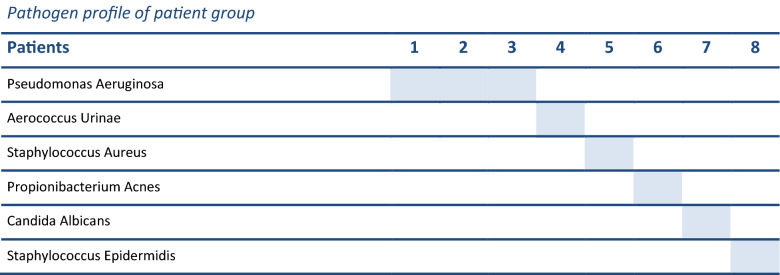
Pathogen profile of the isolated bacteria in our patient group of ISP

### Clinical follow-up (*n* = 5)

The median follow-up time was 13 months (range: 6–30 months). All five patients reported to have consulted at least four doctors before presenting in our department.

1. *Pain*
  The mean preoperative pain (VAS) during walking was 8.6. The mean preoperative VAS for the four analysed categories in the five follow-up patients was 7.2. This value decreased to 2.7 30 days postoperatively, and to 0.4 13 months postoperatively (Fig. 2). The surgical site of all patients was pain free in daily life.

**Fig. 2:**
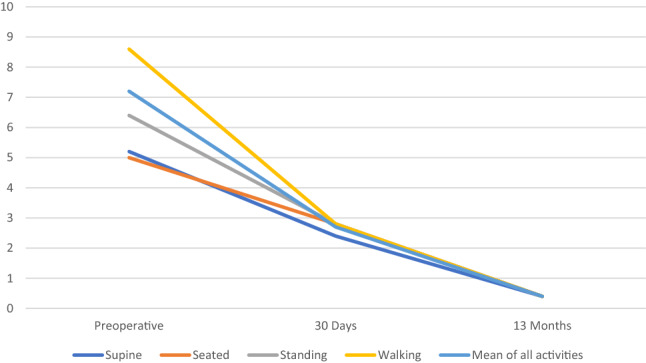
The mean pain scores (VAS) of the five examined patients were obtained through a pain questionnaire on pain during three activities and when rested. A clear reduction of pain can be seen 30 days and 13 months after treatment
2. *Mobility*
  Mobility before the operation was limited with a described range of 0–15 m without walking aids. The most used walking aid was a walker. With walking aids, the patients could walk an average of 100 m (range: 0–400 m). 30 days after surgery, the mean walking distance was 1.2 km, further increasing with time to 3.6 km (range: 2–8 km). Patient 1 was the patient with the longest standing symptoms preoperatively and patient 3 was the oldest patient. Both regained their walking distance slower than the rest of the group. At the time of final follow-up, no patient experienced limitations in mobility attributable to the pubic symphysis (Table 5).

**Table 5:** Walking distance without walking aids on various moments before and after treatment of ISP

Walking distance without walking aids (meter)			
Timepoint	Preop	30 days	13 months
Patient 1	0	0	3000
Patient 2	2	2000	2000
Patient 3	2	25	3000
Patient 4	15	2000	8000
Patient 5	0	2000	2000
3. *Quality of life, infection recurrence and satisfaction*.
  All patients described a serious impact of the disease on quality of life preoperatively. On the four domains questioned, the most affected were in descending order: subjective quality of life, the ability to perform daily tasks, an equal effect on the dependence on nursing staff and psychological disturbances and lastly the effect on the quality of sleep. The mean summated preoperative QoL for the five analysed categories was 2.1; the 30 days postoperative QoL was 3.5 and the 13 months postoperative QoL 4.6. All patients denied a recurrence of infection after treatment. One patient was satisfied, and four patients were very satisfied after treatment.
4. *Clinical examination.*
  The inspection revealed no signs of problematic scar healing or infection. On palpation, the pubic symphysis remained slightly tender with some patients also experiencing discomfort of both pubic rami. Hip flexion and extension were normal in all but one patient, the latter due to osteoarthritis of the hip. Mobility was unrestricted with normal gait in all patients.
5. *Radiology*
  The anteroposterior pelvic X-rays taken at the follow-up were comparable to the postoperative X-rays and showed no significant widening of the symphyseal defect or any new signs of further osteolysis or periosteal reactions. We only recommend anteroposterior pelvic X-rays for the follow-up. We take additional diagnostic measures if a clinical suspicion of ongoing pathology exists after taking standard pelvic X-rays. For bony pathology, we recommend a CT scan, and for soft tissues or infectious relapse an MRI scan is best.

## Discussion

IPS is a painful bacterial infection of the pubic symphysis with a severe impact on the quality of life and mobility. The pubic symphysis is a joint that is not well understood. Its role in causing pain may be overlooked among the broad spectrum of conditions causing subpubic pain. Consequently, as previously described by Sexton et al. in 1993 [14], correct diagnosis and surgical treatment of IPS may be severely delayed. The diagnostic delay in our series was between 30 days and 3 years, resulting in long-term morbidity, loss of mobility and independence, and medical shopping. This is highlighted in literature by the abundance of case reports of patients previously referred to pain clinics, psychiatrists or having chronic opiate abuse [15, 16].

The most valuable information for a possible IPS in our series was previous pelvic surgery of structures of the lesser pelvis. The extent of surgery is not decisive, as other authors also report IPS after minimal invasive surgical procedures: transrectal biopsies, postpartum conditions, robotic-assisted procedures or even simple bladder catheterizations [17–31]. Further, the incidence of IPS is expected to rise in the following decades due to an ageing population with increasing numbers of pelvic procedures and traumatic events in proximity to the symphysis such as prostatectomies, hysterectomies or fragility fractures of the pelvis [32, 33]. Surgeons should have a high index of suspicion when patients present with typical symptoms of IPS and had trauma or previous pelvic surgery. Even when adding other indicators for IPS such as laboratory examinations and radiological workup, a certain diagnosis cannot be made. Some medical conditions are known to increase surgical site infections, e.g. diabetes, obesity and previous malignancy. However, no such medical condition was present in all of our patients. A more detailed analysis of the value of different diagnostic procedures can be found in the Appendix. Amid diagnostic uncertainty, we believe that a clear diagnosis of IPS can only be made through microbiology of surgical tissues.

Many case reports suggest moderate to good outcomes treating IPS with empiric antibiotic therapy monotherapy [27, 34–36]. A partial relapse of symptoms after treatment and sequential antibiotic escalations are frequently mentioned in these cases. A large portion of our cohort had failed previous antibiotic treatments, presenting in our clinic with persistent pain and immobility. Opposing the mentioned studies, isolated antibiotic treatment for IPS was not successful in our patient cohort. After a combination of surgical debridement, application of local and systemic antibiotics, we did not experience relapse of infection. We believe that a careful debridement, preserving the pubic bone and symphyseal ligaments, can improve mobility and lower the incidence of dorsal pelvic instability fractures in osteoporotic individuals [37, 38].

Data on surgical treatment of IPS is scarce. Lavien et al. examined the outcome of pubic bone resection in pubic osteomyelitis due to urosymphyseal fistulas [39]. The researchers described a reduction in pain and chronic narcotic use in a series of 16 patients. Patients were treated using a graded response in which surgery was performed only after initial empiric intravenous antibiotics for 6–8 weeks. The preoperative antibiotic regimen was stated to have poor effect on pain intensity in their group. In contrast, after pubic resection, 15 of 16 patients noted complete resolution of pain. The authors did not state if the diagnosis was confirmed intraoperatively by samples or which pathogens were present. Similarly, it was not mentioned if postoperative antibiotics were used or what effect the procedure had on quality of life and mobility.

Our study has several limitations. First and foremost, the small sample size inhibits statistical analysis and general conclusions cannot be drawn. Due to the retrospective nature of the study, no blinding or randomization was possible, and the availability of preoperative data was limited to the state of the medical records. More research with prospective design and larger sample sizes will be essential to further investigate the diagnostic protocol and optimal treatment of IPS.

## Conclusion

We consider open surgery as the golden standard to treat IPS. Careful surgical debridement without damaging the symphyseal ligaments in combination with local antibiotics is the most important step of therapy. This should be followed by an antibiotic regimen adjusted to the pathogen resistance profile. Patients should be evaluated multidisciplinarily by the pelvic surgery team and infectious disease experts. Surgical treatment of septic pubic symphysitis provides a safe and effective therapy with excellent objective and subjective outcomes.

## Appendix: Laboratory examination and radiological workup

Laboratory examinations are used commonly as indicator for an ongoing infection. However, especially with chronic infections or previous antibiotic treatment, examinations such as leucocyte count (Lc) and C-reactive protein (CRP) were not helpful in assuming the extent of the pubic infection in our patient group, occasionally even being misleading. Also, the pathogen spectrum may play a role, *Pseudomonas aeruginosa* or S*. epidermidis* are less virulent germs with a low systemic inflammation response. Finally, the restricted blood perfusion of the pubic joint space can keep the infection relatively isolated from the bloodstream [40]. This may also be a reason for suboptimal results with systemic monotherapy with corticosteroids or intravenous antibiotics, reported by other authors [15, 39]. The unspectacular intraoperative findings in the absence of pus, as well as the clinical absence of fever, malaise, infectious spreading and sepsis, which was observed in our patient series, may also be explained by the restricted blood supply to the symphysis pubis joint space.

Radiological workup of IPS is challenging. McPhee et al. noted that no certain diagnosis can be made by plain radiography, computed tomography or three-phase 99mTc bone scanning [41]. Moreover, a recent review quoted 63% of plain radiographs to be negative in confirmed pubic bone osteomyelitis, despite progression with urinary fistula formation [42]. Nevertheless, plain radiographs still offer fast, cheap and valuable information towards differentiating IPS from other diagnoses such as pubic bone fractures, symphyseal disruption and chronic instability. In our experience, further diagnostics such as CT and MRI are necessary to evaluate the pubic symphysis. Signs of IPS include high signal intensity on T2-weighted and low signal intensity on T1-weighted images, reflecting joint space signal intensity, due to oedema of the surrounding tissues and exudate in the joint space [43]. Distinguishing aseptic osteitis pubis and IPS by MRI can be challenging in early phases. Although recent efforts were made to radiologically classify aseptic osteitis pubis, no hints were given to differentiate from IPS [44].

## Footnotes

1 Ethics commission of the state chamber of medicine of Rhineland-Palatinate (Ref. 837.140.17 (10,974)).

2 ICD (International statistical Classification of Diseases)—codes searched: S31.1, M13.15, M13.85, M25.55, M85.3, M86.25, M86.15, M86.05, M86,45, M86.55, M86.65, O26.719 and OPS (Operations and Procedure Code)-Code 5–800.3F.

## References

[1] Jorgensen SG, Oberg S, Rosenberg J (2019). Treatment of longstanding groin pain: a systematic review. Hernia J Hernias Abdom Wall Surg.

[2] Cardoso L, Alves P, Santos F (2017). Septic arthritis of the pubic symphysis. Case Rep.

[3] Lentz SS (1995). Osteitis pubis: a review. Obstet Gynecol Surv.

[4] Andrews SK, Carek PJ (1998). Osteitis pubis: a diagnosis for the family physician. J Am Board Family Pract.

[5] Ross JJ, Hu LT (2003). Septic arthritis of the pubic symphysis: review of 100 cases. Medicine.

[6] McHenry MC, Alfidi RJ, Wilde AH, Hawk WA (1975). Hematogenous osteomyelitis; a changing disease. Cleve Clin Q.

[7] Coventry MB, Mitchell WC (1961). Osteitis pubis: observations based on a study of 45 patients. JAMA.

[8] Burns JR, Gregory JG (1977). Osteomyelitis of the pubic symphysis after urologic surgery. J Urol.

[9] Beer E (1924). Periostitis of symphysis and descending rami of pubis following suprapubic operations. Int J Med Surg.

[10] Andrews JA, Rizzato Lede D, Senderovsky M, Finn BC, Emery N, Bottaro F, Bruetman JE, Young P (2012). Septic arthritis of the pubic symphysis in two athletes. Medicina.

[11] Gamble JG, Simmons SC, Freedman M (1986). The symphysis pubis. Anatomic and pathologic considerations. Clin Orthop Relat Res.

[12] Speer LM, Mushkbar S, Erbele T (2016). Chronic pelvic pain in women. Am Fam Physician.

[13] Stamey TA (1981). Prostatitis. J R Soc Med.

[14] Sexton DJ, Heskestad L, Lambeth WR, McCallum R, Levin LS, Corey GR (1993). Postoperative pubic osteomyelitis misdiagnosed as osteitis pubis: report of four cases and review. Clin Infect Dis.

[15] Choi H, McCartney M, Best TM (2011). Treatment of osteitis pubis and osteomyelitis of the pubic symphysis in athletes: a systematic review. Br J Sports Med.

[16] Adams RJ, Chandler FA (1953). Osteitis pubis of traumatic etiology. J Bone Jt Surg Am.

[17] Adam C, Graser A, Koch W, Trottmann M, Rohrmann K, Zaak D, Stief C (2006). Symphysitis following transrectal biopsy of the prostate. Int J Urol.

[18] Bensouda A, Kulisa M, Poissonnier L, Badet L, Colombel M, Martin X, Gelet A (2009). Fassi-Fehri H (2009) [Bone-anchored suburethral sling complicated by pubic osteomyelitis: a case report]. Prog Urol.

[19] Denes E, Camilleri Y, Fiorenza F (2015). Martin C (2015) First case of osteomyelitis due to Erysipelothrix rhusiopathiae: pubic osteomyelitis in a gored farmer. Int J Infect Dis.

[20] Cosma S, Borella F, Carosso A, Ingala A, Fassio F, Robba T, Maina A, Bertero L, Benedetto C (2019). Osteomyelitis of the pubic symphysis caused by methicillin-resistant Staphylococcus aureus after vaginal delivery: a case report and literature review. BMC Infect Dis.

[21] Degheili JA, Mansour MM, Nasr RW (2018). Symphysis pubis osteomyelitis: an uncommon complication after robotic assisted radical prostatectomy-case description with literature review. Case Rep Urol.

[22] Fukui S, Iemura Y, Matsumura Y, Kagebayashi Y, Samma S (2019). Pubic bone osteomyelitis in patient with prostate cancer after robot-assisted laparoscopic prostatectomy; a case report. Hinyokika kiyo Acta urologica Japonica.

[23] Gerullis H, Eitzen A, Uphoff J, Daaboul F, Chavan A, Ermert L, Wawroschek F, Winter A (2017). Recurrent symphysitis culminating in pelvic ring fracture after hyperextended transurethral prostate resection and vaporization with symphysis erosion: a case report. J Medi Case Rep.

[24] Goldberg RP, Tchetgen MB, Sand PK, Koduri S, Rackley R, Appell R, Culligan PJ (2004). Incidence of pubic osteomyelitis after bladder neck suspension using bone anchors. Urology.

[25] Graham CW, Dmochowski RR, Faerber GJ, Clemens JQ, Westney OL (2002). Pubic osteomyelitis following bladder neck surgery using bone anchors: a report of 9 cases. J Urol.

[26] Haensel A, Naumann G, Hohle P, Rommens PM, Koelbl H (2007). Osteomyelitis following a transobturator sling (TVT-O). BJOG Int J Obst Gynaecol.

[27] Hocedez C, Pelissier A, Mosbah R, Raimond E, Gabriel R, Graesslin O (2015). Septic arthritis of the pubic symphysis during pregnancy. Gynecol Obst Fert.

[28] Kroft J, Kung RC (2009). Postoperative pubic symphysis osteomyelitis after laparoscopic two-team sling with anterior and posterior colporrhaphy. J Minim Invas Gynecol.

[29] Naqvi N, Naqvi R, Wong C, Pearce S (2008). A novel observation of pubic osteomyelitis due to *Streptococcus viridans* after dental extraction: a case report. J Med Case Rep.

[30] Robison CM, Gor RA, Metro MJ (2013). Pubic bone osteomyelitis after salvage high-intensity focused ultrasound for prostate cancer. Curr Urol.

[31] Stern JA, Clemens JQ (2003). Osteomyelitis of the pubis: a complication of a chronic indwelling catheter. Urology.

[32] Rommens PM, Hofmann A (2013). Comprehensive classification of fragility fractures of the pelvic ring: recommendations for surgical treatment. Injury.

[33] Bugeja S, Andrich DE, Mundy AR (2016). Fistulation into the pubic symphysis after treatment of prostate cancer: an important and surgically correctable complication. J Urol.

[34] Hartshorn S, Davies K, Anderson JM (2009). Septic arthritis of the pubic symphysis in an 11-year-old boy. Pediatr Emerg Care.

[35] Johnstone A, Gough A (2011). Pubic rami and sacral insufficiency fractures resulting in osteomyelitis, vulval abscess, discharging bone fragments and pelvic instability. JRSM Short Rep.

[36] Konik E, Bauer B, Lee M (2011). 64-year-old male with septic arthritis of the pubic symphysis. Clin Pract.

[37] Lau TW, Leung F (2010). Occult posterior pelvic ring fractures in elderly patients with osteoporotic pubic rami fractures. J Orthopaed Surg (Hong Kong).

[38] Rommens PM, Wagner D, Hofmann A (2012). Surgical management of osteoporotic pelvic fractures: a new challenge. Eur J Trauma Emerg Surg Off Publ Eur Trauma Soc.

[39] Lavien G, Chery G, Zaid UB, Peterson AC (2017). Pubic bone resection provides objective pain control in the prostate cancer survivor with pubic bone osteomyelitis with an associated urinary tract to pubic symphysis fistula. Urology.

[40] Vandijck DM, Hoste EA, Blot SI, Depuydt PO, Peleman RA, Decruyenaere JM (2007). Dynamics of C-reactive protein and white blood cell count in critically ill patients with nosocomial Gram positive vs. Gram negative bacteremia: a historical cohort study. BMC Infect Dis.

[41] McPhee E, Eskander JP, Eskander MS, Mahan ST, Mortimer E (2007). Imaging in pelvic osteomyelitis: support for early magnetic resonance imaging. J Pediatr Orthop.

[42] Sexton SJ, Lavien G, Said N, Eward W, Peterson AC, Gupta RT (2019). Magnetic resonance imaging features of pubic symphysis urinary fistula with pubic bone osteomyelitis in the treated prostate cancer patient. Abdom Radiol (NY).

[43] Santiago Restrepo C, Gimenez CR, McCarthy K (2003). Imaging of osteomyelitis and musculoskeletal soft tissue infections: current concepts. Rheum Dis Clin North Am.

[44] Gaudino F, Weber MA (2019). Osteitis pubis or symphysitis pubis. Der Radiologe.

